# Length of exposure to long working hours and night work and risk of sickness absence: a register-based cohort study

**DOI:** 10.1186/s12913-021-07231-4

**Published:** 2021-11-05

**Authors:** Laura Peutere, Tom Rosenström, Aki Koskinen, Mikko Härmä, Mika Kivimäki, Marianna Virtanen, Jenni Ervasti, Annina Ropponen

**Affiliations:** 1 School of Educational Sciences and Psychology, University of Eastern Finland, Joensuu, Finland; 2 Department of Psychology and Logopedics, Faculty of Medicine, University of Helsinki, Helsinki, Finland; 3 Finnish Institute of Occupational Health, Helsinki, Finland; 4 Clinicum, Faculty of Medicine, University of Helsinki, Helsinki, Finland; 5 Department of Epidemiology and Public Health, University College London, London, UK; 6 Division of Insurance Medicine, Department of Clinical Neuroscience, Karolinska Institutet, Stockholm, Sweden

**Keywords:** Sickness absence, Night work, Shift work, Exposure time, Nurses, Working hours

## Abstract

**Background:**

There is inconsistent evidence that long working hours and night work are risk factors for sickness absence, but few studies have considered variation in the length of exposure time window as a potential source of mixed findings. We examined whether the association of long working hours and night work with sickness absence is dependent on the length of exposure to the working hour characteristics.

**Methods:**

We analysed records of working hours, night work and sickness absence for a cohort of 9226 employees in one hospital district in Finland between 2008 and 2019. The exposure time windows ranged from 10 to 180 days, and we used Cox’s proportional hazards models with time-dependent exposures to analyse the associations between working-hour characteristics and subsequent sickness absence.

**Results:**

Longer working hours for a period of 10 to 30 days was not associated with the risk of sickness absence whereas longer working hours for a period of 40 to 180 days was associated with a lower risk of sickness absence. Irrespective of exposure time window, night work was not associated with sickness absence.

**Conclusions:**

It is important to consider the length of exposure time window when examining associations between long working hours and sickness absence, whereas the association between night work and sickness absence is not similarly sensitive to exposure times.

**Supplementary Information:**

The online version contains supplementary material available at 10.1186/s12913-021-07231-4.

## Introduction

Long working hours and shift work are important occupational risk factors, particularly in sectors such as health care, where exposure to these working-hour characteristics is often unavoidable [1, 2]. Both long working hours and shift work may lead to poor health by limiting time for sleep and recovery and increasing unhealthy habits such as night time eating or reduced physical activity [3–6]. Shift work, particularly at night, also disrupts the circadian rhythm, which may aggravate health problems [4].

Although long working hours are related to increased health problems, the associations with sickness absence (SA) seem to be more complex. While some studies show a higher risk of SA among people who work long hours [7–10], others have reported that long working hours are associated with a lower risk of SA [7, 11–13]. The direction of the associations may also vary within the same study depending, for example, on the specific measures of working hour characteristics and SA. Similarly, shift and night work have been associated with both higher [8, 14–16] and lower [16] risks of SA. Furthermore, some studies have found no association between night work and SA [8, 13, 17]. These inconsistent findings may be at least partly explained by a healthy worker effect (healthier employees are more capable of working extended hours and working at night than less healthy employees) or differences in work attendance motivation or working conditions, such as resources and demands [12]. A further possibility is the role of variation in the lengths of exposure time windows to working hour characteristics; over prolonged periods, the harmful effects of these occupational risk factors may accumulate. Furthermore, calling in sick may be used as a strategy to recover from prolonged exposure to long work shifts, repeated night work or insufficient rest between shifts [8, 10, 17]. Prior studies have measured the exposure to long working hours and night work using different exposure time windows ranging from 7 days [10, 15] to 4 years [16], a potential source of inconsistencies between studies**.**

In this study, the aim was to examine whether the association of long working hours and night work with SA is dependent on exposure time window.

## Method

### Sample

The data were retrieved from a payroll-based, employer-owned register from the Hospital District of Southwest Finland using the shift scheduling program Titania®. The data included information on employees’ planned and actual working hours, SA days, rest days, annual holidays, and other paid and unpaid leave for 2008–2019 [18]. As a main rule, each employee had at least one row in the raw data for each calendar day. However, employees with temporary jobs or other breaks in their job contracts may have had breaks of various lengths in the register data.

For the purposes of this study, the data were organized to the accuracy of calendar days. First, we first calculated the number of working hours and the number of night work hours per shift and recorded these hours in the calendar day when each shift ended. SA days were recorded from the first day of absence. Exposure to long working hours and night work of each employee was assessed from the first day of employment contract (or 1 January 2008 if the contract started before 2008) until his/her first SA day (the length of the SA was not considered); the end of his/her employment contract or other break in employment lasting at least 2 days; the start of family leave (for example parental leave, maternity leave) or unpaid leave (for example due to studies) of any length, or the end of 2019 – whichever came first. The follow-up periods were not ended or censored due to normal rest days or vacations. The data included information on employees’ contract types. We only included employees who – for the most days in their follow-ups – were employed based on certain period-based, day^1^ or shift work contract. This restriction excluded physicians and office workers who, according to the available data description, have a different contract type. Information on physicians’ on-call work was not available from the register, and office workers do not work shifts, and therefore, were not included in the analyses. To compare different exposure time windows within the same sample, the data were further restricted to employees with a given minimum number of consecutive days without SA, other leave, or breaks. For the analyses based on the exposure time window of 30 days at maximum, at least 31 days of data was required on an employee (i.e. the length of the exposure time + an additional day of data). Each employee was also required to have at least 2 actualized work shifts during these first 30 days of the follow-up. Accounting for these conditions, the number of employees remaining in the sample was 9226. Additional analyses were conducted for exposure time windows of 60, 90, 120, 150 and 180 days at maximum. Following the same logic, the minimum amount of data required for each employee was the maximum number of days + 1 of each sample (and at least two actualized work shifts during this period), resulting in sample sizes of 7859, 6448, 5689, 4961 and 4268, respectively. However, multiple shorter exposure time windows were also analysed using an identical sample. These different restrictions for samples allowed us to further increase the maximum exposure time while still analysing the same individuals within each sample. Study protocol and overall design of the analyses were defined before conducting any analyses, but the length of maximum exposure time windows were determined during data analysis, based on the number of employees who remained in each sample.

### Working-hour characteristics

The average number of working hours was calculated separately for each exposure time window for each employee in each sample. These moving time averages were calculated as the sum of the working hours over the calendar days divided by the length of the exposure time window, i.e., by the number of calendar days over which the working hours were averaged. The moving averages were used as time-dependent exposure variables for regression models so that for each day in the follow-up, they represented the average length of working hours in the preceding days for a given employee.

The time averages of night work hours (=hours worked between 11 pm and 6 am) were also recorded as time-dependent exposure variables for each time window.

### Statistical analyses

We used Cox’s proportional hazards model with time-dependent exposure variables. Risk of SA was predicted based on the time averages of working hours and night work hours in the preceding period (for example, previous 10, 30 or 180 days). In the analyses based on each sample, the follow-ups always start from the *T*th + 1 observed day for an employee, where *T* is the size of the maximum time window. For example, in the analyses based on sample that included employees who could be followed up at least 30 + 1 calendar days (that is, the sample with the maximum time window of 30 days) the follow-ups start from the 31th day, while for employees who could be followed up at least 60 + 1 day, the follow-ups start from the 61th day, etc. (see also Additional file 1: Appendix Fig. A1). The results are presented for 10-day increments in moving exposure time windows based on the samples with maximum exposure time windows of 30 and 60 days (starting from 10 days), for 20-day increments for 90 days (starting from 10 days), for 20-day increments for 120 days (starting from 20 days), for 30-day increments for 150 days (starting from 30 days) and for 40-day increments for 180 days (starting from 20 days). The results for working hours were adjusted for sex and age, and the results for night work hours were adjusted for sex, age, and overall working hours. Age was included as a continuous time-variable indicating employees’ age in each day of the follow-up period. As night work is relatively uncommon, we also run sensitivity analyses among employees who had any night work during their follow-ups. The Cox models were estimated using the coxph function of the Survival package in R [19].

## Results

Table 1 shows descriptive information on the samples and variables for exposure time windows of different lengths: 30, 60, 90, 120, 150 and 180 days. The proportions of women (89–91%) and employees working full time (91–93%) were similar across the samples. The proportion of employees with a day work contract and the mean age of employees were slightly higher in analysis of longer maximum exposure time windows: the longer the maximum exposure time window of the sample, the greater the proportion of follow-ups that ended to an SA event was.

**Table 1:** Details of the six samples based on exposure time windows of 30–180 days at maximum

	30 days	60 days	90 days	120 days	150 days	180 days
Number of employees	9226	7859	6448	5689	4961	4268
Person days	2,302,550	2,058,224	1,847,231	1,667,510	1,506,785	1,369,733
	%	%	%	%	%	%
Women	89.2	89.9	90.6	90.6	90.7	90.9
Day work contract ^a b^	37.8	38.7	39.7	40.2	41.0	41.6
Shift work contract ^a b^	61.7	61.0	59.9	59.5	58.6	58.0
No night shifts during the follow-up	54.9	53.1	53.2	52.7	53.1	53.0
Full time^a^	92.9	92.3	91.5	91.1	91.1	92.6
Follow-up ending in sickness absence	61.8	64.5	72.2	76.6	77.1	77.5
	mean (sd)	mean (sd)	mean (sd)	mean (sd)	mean (sd)	mean (sd)
Length of follow-up (days)	279.6 (367.4)	321.9 (383.6)	376.5 (403.6)	413.1 (416.3)	453.7 (431.1)	500.9 (447.3)
Age^a^	38.3 (12.6)	39.7 (12.5)	41.9 (11.8)	42.9 (11.6)	43.4 (11.4)	43.8 (11.3)
Working hours^c^	4.3 (1.3)	4.3 (1.0)	4.3 (0.8)	4.3 (0.8)	4.3 (0.7)	4.3 (0.7)
Night work hours^c^	0.3 (0.5)	0.3 (0.5)	0.3 (0.5)	0.3 (0.5)	0.3 (0.5)	0.3 (0.5)

^a^ Measured at the start of the follow-up

^b^ Employees were included based on the most common contract type during their follow-ups. In the first day of the follow-up, small proportion of them had another contract type

^c^ Distribution (overall) of the time-averages for the longest exposure time window of each sample; includes days off. For example, 40 h of work within a seven-day period corresponds to an average of 5.7 h of work per calendar day

The distributions of working hour characteristics were quite similar or identical for the maximum exposure time windows across all the samples (Table 1). More than half (53–55%) of the employees did not work any night shifts during their follow-up.

**Fig. 1:**
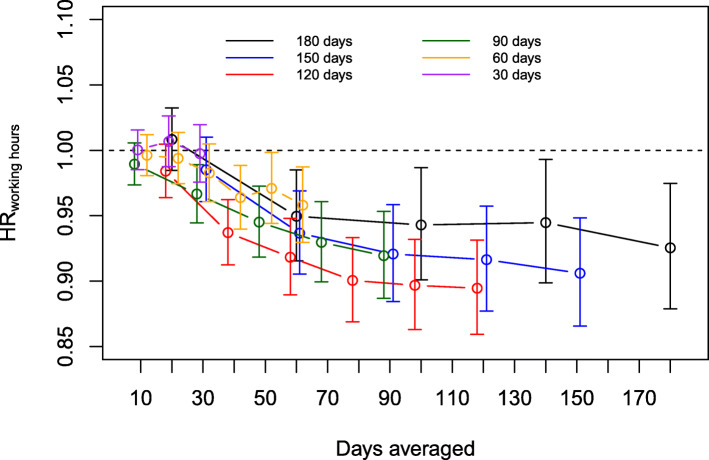
Associations between time-averaged working hours (proportional hazard ratios [HR]) and immediately following SA events in analysis runs based on samples for exposure time windows of 30 to 180 days at maximum. Within each maximum exposure time window (shown in a distinct colour), the sample stayed constant and only the exposure time window (x-axis) varied. The results are adjusted for age and sex

Apart from one exception, longer average daily working hours were not associated with a risk of SA, especially for short exposure time windows ranging from 10 to 30 days (Fig. 1 and Additional file 1: Appendix Table A1). The highest HRs (1.01 95% CI 0.99–1.03 and 1.01 95% CI 0.98–1.03) of longer working hours as a risk factor for SA were in the exposure time window of 20 days in the samples based on the maximum time windows of 30 and 180 days. For exposure time windows of 40 days or more, the HR for SA and their confidence intervals fell below 1, indicating that longer working hours were associated with a lower risk of SA. The lowest HR of longer working hours as a risk factor for SA indicated that each one-hour increase in average working hours within 120 days was associated with 11% lower risk of SA (HR 0.89, 95% CI 0.86–0.93). Figure 1 also shows that the associations followed a similar pattern with overlapping confidence intervals in all six samples.

**Fig. 2:**
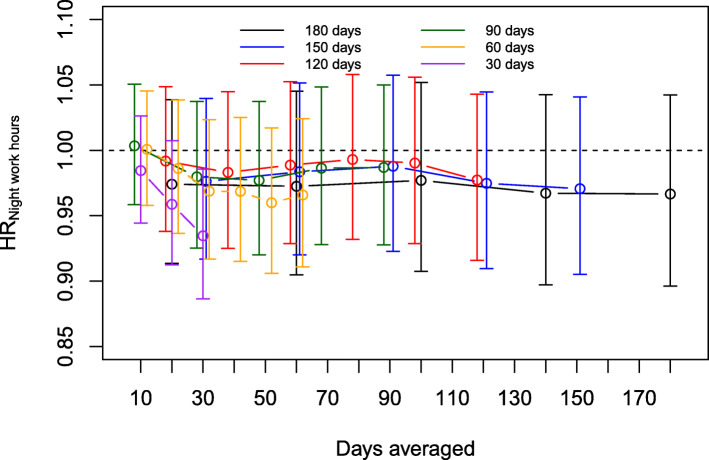
Associations between time-averaged night work hours (proportional hazard ratios [HR]) and immediately following an SA event in analysis runs based on samples for exposure time windows of 30 to 180 days at maximum. Within each maximum exposure time window (shown in a distinct colour), the sample stayed constant and only the exposure time window (x-axis) varied. The results are adjusted for time-averaged working hours, age and sex

Regarding night work hours, the HRs stayed consistently below 1 in most of the exposure time windows (Fig. 2 and Additional file 1: Appendix Table A1). Longer average night work hours were, however, associated with a lower risk of SA only in the exposure time window of 30 days in the sample based on maximum time window of 30 days (0.93, 95% CI 0.89–0.99).

The pattern of associations was to a large extent similar in all six samples with overlapping confidence intervals.

Compared to the main analyses, the HRs for SA were higher and mainly above 1 when the samples were restricted to employees who had any night work during their follow-ups (Additional file 1: Appendix Fig. A2). However, night work hours were not associated with SA in these samples either.

## Discussion

This cohort study of register-based data on working-hour characteristics of employees in a hospital district in Finland examined whether the length of exposure time window affects the associations between working hours, night work and SA. The results show that the associations depended on the length of exposure time window; specifically, the association between longer working hours and lower risk of SA appeared to emerge when considering exposure time windows of 40 days or more. Night work, in turn, was predominantly not associated with SA irrespective of the exposure time window.

In recent years, studies have increasingly utilized routinely collected administrative data on working hours in the health care sector to investigate the associations between working hours and SA [8–11, 13–15, 17]. However, the lengths of exposure periods used for previous studies vary considerably, potentially leading to mixed associations with SA. This study contributes to this branch of research by providing comparisons of exposure time windows ranging from 10 to 180 days and assessing the first incidence of SA.

We differentiated between two characteristics of daily working hours: the number of working hours and the number of night work hours. For exposure time windows of 10 to 30 days, longer working hours were predominantly not associated with SA. However, for exposure time windows of ≥40 days, longer working hours were associated with a lower risk of SA. Regarding short exposure time windows of 10 to 30 days, our (null) findings contrast with earlier positive associations between long working hours and SA in short < 30 days windows [8, 10], but are in line with one study showing a null association [17]. The differences in results may relate to the ways in which working hour characteristics have been measured, as well as differences in study designs. For example, a prior study [8] based on a larger data set from Finnish hospitals was based on a case-crossover design and focused on employees with first incident short (1–3 days) SA, whereas our study considered SA of any length. Regarding longer exposure time windows, our results are at least partly in line with studies in which exposure to long working hours over 3 months [13] and a year [7, 11] were associated with a lower risk of sickness absence, and contradict with the results of two studies [7, 8]. For example, a high number of long shifts and weeks within a year was associated with an increased risk of long-term sickness absence in Finland, while reversed associations were found in Denmark [7].

Our study also showed that night work was not associated with SA, irrespective of exposure time window. This result is in line with prior studies [8, 13, 17], and in contrast with one study that indicated higher risk of SA [8]. The differences in results may be explained by measures of night work. For example, in the above-mentioned Finnish study the proportion of night shifts of all shifts was not associated with SA, while consecutive night shifts increased the risk of SA [8].

Given that long working hours or night work in short-term were not associated with increased risk of SA, the results of this study do not support the notion that workers tend to call in sick as a strategy to recover from prolonged exposure to long work shifts, repeated night work or insufficient rest between shifts [8, 10, 17]. The observed lower SA risk associated with long compared to standard working hours in extended exposure time windows may be attributable to health-related selection: the healthiest employees can work long hours over prolonged periods while those with health problems may choose to work standard or reduced hours. It is also noteworthy that long working hours over an extended period is an average measure. In a long exposure time window, there might be both peaks of extremely long working hours over a short period and standard hours over a longer period.

The lack of association between night work and SA could also be explained by health selection effects. It must also be noted that more than half of the employees in this study did not work night shifts at all. Employees with health problems may avoid night shifts or quickly change from night work to day work. According to an EU directive [20], employees who suffer from health problems as a result of night work should not be required to work night shifts. There may also be other differences between employees who prefer night shifts and those who do not work nights.

### Strengths and limitations

The strength of this study was the large, objective data set on working hours for an 11-year period, which meant that it had no limitations related to memory bias, nonresponse or loss of follow-up data [18]. The payroll-based data also provided the opportunity to analyse daily working-hour characteristics, which would not have been possible in a study based on self-reporting.

In our study, we compared different exposure time windows both within and between samples. The largest sample included employees who could be followed up at least 31 days, and the sample sizes decreased substantially as the length of the maximum exposure time window increased. The composition of employees in the different samples varied in age and contract type. Younger employees and employees on shift-work contracts may have had more interruptions in their employment and therefore have been excluded from our samples of longer exposure time windows that required more follow-up data. Another aspect that may play a role is health-related selection. It can be assumed that employees with poor health and frequent SA might have been excluded from the samples for longer exposure time windows. However, the characteristics of the samples were similar and the associations found between working-hour characteristics and SA exhibited very similar patterns with overlapping confidence intervals, suggesting that our findings are unlikely to be explained entirely by health-related selection. Also, the comparisons of the different exposure time windows within each sample were based on the same individuals.

The main limitation of this study was the lack of certain potentially relevant control variables, such as job titles or education levels of employees. In Finland, practical nurses are required to have at least completed vocational education while nurses are required to have a degree from a university of applied sciences. In the Hospital District of Southwest Finland, only 8% of all employees are not educated beyond primary level or are students [21]. Prior studies of Finnish hospital districts show that the associations between working-hour characteristics and SA are similar for employees with different job titles and in different hospital districts [8], age groups [9] and when adjusting for organizational units [7].

This study followed up employees from the first day of their employment contract, or 1 January 2008, whichever was latest, until first SA or end of follow, whichever came first. The observations of the participants were not censored at their vacations although the risk for calling in sick is lower – but still possible – during those days. Vacations are a normal part of working times and especially long follow-ups, such as 6 months, are likely to include vacations. By excluding participants with SA during the set exposure time windows, we were unable to control for SA prior to the start of the study or other health or motivational differences between employees. It is therefore possible that employees with better health and stronger attendance motivation both worked longer hours and more night shifts and were less likely to call in sick. However, we have no reason to believe that the possible confounding in the associations due to a lack of control variables would vary because of different length exposure time windows within the same sample. Thus, the data can be considered reliable for these comparisons.

In this study we focused on first incidents of SA of any length; however, analyses based on recurrent or across various length periods of SA may show different results [14, 16]. It is also uncommon for nurses to regularly work very long-hour weeks in Finland due to work time regulations [22], although we believe that our results are generalizable to Nordic countries with similar welfare societies. Future studies are needed to replicate our analysis for other samples, occupational groups and longer exposure times and adjust for individual- and workplace-related factors.

## Conclusions

Exposure to long working hours for a period of 10 to 30 days was not associated with a risk of SA, but an association between longer working hours and lower risk of SA emerged when considering exposure time windows of 40 days or more. Longer night work hours were not associated with SA irrespective of exposure time window. In all, this study highlights the importance of exposure time windows when analysing and interpreting the associations between working hours and SA.

## Supplementary Information

**Additional file 1.**

## Data Availability

The data sets generated and analysed for the current study are not publicly available. According to the General Data Protection Regulation, this type of sensitive data can only be made available to researchers who, after a legal review, meet the criteria for access to this type of sensitive and confidential data. Readers may contact the last author regarding these details.

## Abbreviation

SA: Sickness absence

## Footnotes

1 Employees with a day work contract may also occasionally work in shifts.
