# Supplementary material for: Length of exposure to long working hours and night work and risk of sickness absence: a register-based cohort study
Source: BMC Health Serv Res. 2021 Nov 5;21:1199. doi: 10.1186/s12913-021-07231-4 (PMC8571875; doi:10.1186/s12913-021-07231-4)
Supplement: Supplementary file 1 — Additional file 1. [file 12913_2021_7231_MOESM1_ESM.docx]

Appendix

Figure A1. An example of the follow-up of an employee in the sample based on the maximum exposure time window of 30 days

| Appendix Table A1. Hazard ratios (HR) for sickness absence as a function of time-averaged working hours and night work hours, stratified by sample and exposure time window. | | | | | | | |
| --- | --- | --- | --- | --- | --- | --- | --- |
| Sample | Exposure time window (days) | Working hours¹ | | | Night work hours² | | |
|  |  | HR | 95% CI | | HR | 95% CI | |
|  | 10 | 1.00 | 0.99 | 1.02 | 0.98 | 0.94 | 1.03 |
| 30 days | 20 | 1.01 | 0.99 | 1.03 | 0.96 | 0.91 | 1.01 |
|  | 30 | 1.00 | 0.98 | 1.02 | **0.93** | **0.89** | **0.99** |
|  |  |  |  |  |  |  |  |
| 60 days | 10 | 1.00 | 0.98 | 1.01 | 1.00 | 0.96 | 1.05 |
|  | 20 | 0.99 | 0.97 | 1.01 | 0.99 | 0.94 | 1.04 |
|  | 30 | 0.98 | 0.96 | 1.00 | 0.97 | 0.92 | 1.02 |
|  | 40 | **0.96** | **0.94** | **0.99** | 0.97 | 0.92 | 1.03 |
|  | 50 | 0.97 | 0.94 | 1.00 | 0.96 | 0.91 | 1.02 |
|  | 60 | **0.96** | **0.93** | **0.99** | 0.97 | 0.91 | 1.02 |
|  |  |  |  |  |  |  |  |
| 90 days | 10 | 0.99 | 0.97 | 1.01 | 1.00 | 0.96 | 1.05 |
|  | 30 | **0.97** | **0.94** | **0.99** | 0.98 | 0.93 | 1.04 |
|  | 50 | **0.95** | **0.92** | **0.97** | 0.98 | 0.92 | 1.04 |
|  | 70 | **0.93** | **0.90** | **0.96** | 0.99 | 0.93 | 1.05 |
|  | 90 | **0.92** | **0.89** | **0.95** | 0.99 | 0.93 | 1.05 |
|  |  |  |  |  |  |  |  |
| 120 days | 20 | 0.98 | 0.96 | 1.00 | 0.99 | 0.94 | 1.05 |
|  | 40 | **0.94** | **0.91** | **0.96** | 0.98 | 0.93 | 1.04 |
|  | 60 | **0.92** | **0.89** | **0.95** | 0.99 | 0.93 | 1.05 |
|  | 80 | **0.90** | **0.87** | **0.93** | 0.99 | 0.93 | 1.06 |
|  | 100 | **0.90** | **0.86** | **0.93** | 0.99 | 0.93 | 1.06 |
|  | 120 | **0.89** | **0.86** | **0.93** | 0.98 | 0.92 | 1.04 |
|  |  |  |  |  |  |  |  |
| 150 days | 30 | 0.99 | 0.96 | 1.01 | 0.98 | 0.92 | 1.04 |
|  | 60 | **0.94** | **0.91** | **0.97** | 0.98 | 0.92 | 1.05 |
|  | 90 | **0.92** | **0.88** | **0.96** | 0.99 | 0.92 | 1.06 |
|  | 120 | **0.92** | **0.88** | **0.96** | 0.97 | 0.91 | 1.04 |
|  | 150 | **0.91** | **0.87** | **0.95** | 0.97 | 0.91 | 1.04 |
|  |  |  |  |  |  |  |  |
| 180 days | 20 | 1.01 | 0.98 | 1.03 | 0.97 | 0.91 | 1.04 |
|  | 60 | **0.95** | **0.92** | **0.99** | 0.97 | 0.90 | 1.05 |
|  | 100 | **0.94** | **0.90** | **0.99** | 0.98 | 0.91 | 1.05 |
|  | 140 | **0.94** | **0.90** | **0.99** | 0.97 | 0.90 | 1.04 |
|  | 180 | **0.93** | **0.88** | **0.97** | 0.97 | 0.90 | 1.04 |
| ¹ Adjusted for age and sex. ² Adjusted for age and sex, and the time-averaged working hours. Statistically significant HR with 95%CI in boldface. | | | | | | | |


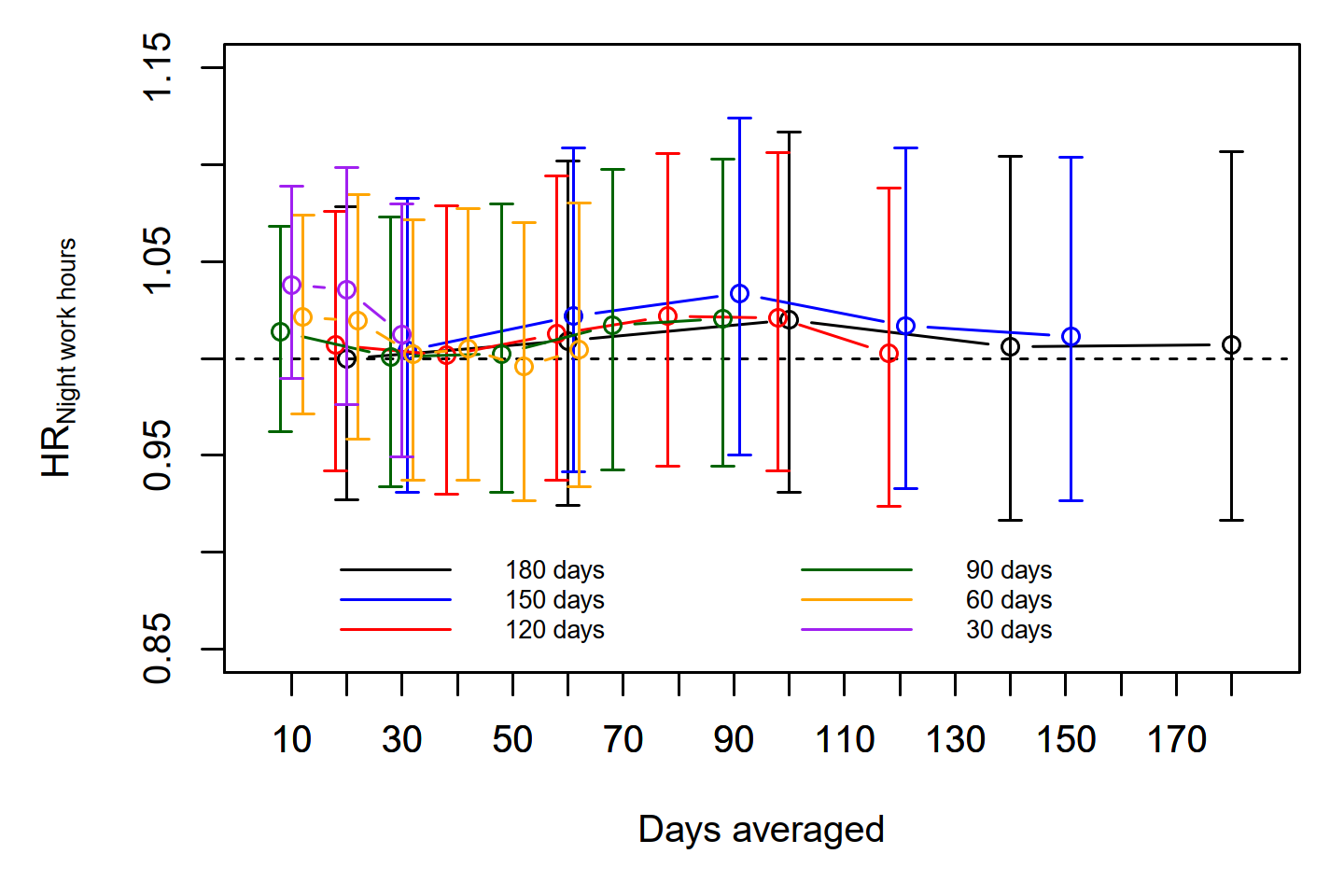


Figure A2. Associations between night work hours (proportional hazard ratios [HR]) and immediately following an SA event in analysis runs based on samples for exposure time windows of 30 to 180 days at maximum. Includes only employees who had any night work during their follow-ups. Within each maximum exposure time window (shown in a distinct colour), the sample stayed constant and only the exposure time window (x-axis) varied. The results are adjusted for time-averaged working hours, age and sex.
